# Paternal factors in spontaneous first trimester miscarriage

**DOI:** 10.12669/pjms.293.3388

**Published:** 2013

**Authors:** Riffat Jaleel, Ayesha Khan

**Affiliations:** 1 Dr. Riffat Jaleel, MBBS, FCPS, Assistant Professor, Obstetrics & Gynaecology, Unit II, Dow Medical College & Civil Hospital, Dow University of Health Sciences, Karachi, Pakistan.; 2 Ayesha Khan, MBBS, FRCOG, Professor, Obstetrics & Gynaecology, Unit II, Dow Medical College & Civil Hospital, Dow University of Health Sciences, Karachi, Pakistan.

**Keywords:** Paternal age, Spontaneous miscarriage, Drug abuse, Male genital tract infection

## Abstract

***Objectives*** ***:*** To determine whether paternal factors i.e., age, tobacco use and genital tract infection increase the risk for spontaneous first trimester miscarriage.

***Methodology*** ***: ***This case control study was conducted in the Department of Obstetrics & Gynaecology, Unit V / IV, Dow Medical College & Lyari General Hospital, Dow University of Health Sciences, Karachi, Pakistan. Duration of study was two and half years, from Nov, 2007 to Apr, 2010. Inclusion criteria were pregnant women with age 20 – 35 years irrespective of parity. Exclusion criteria were known medical illness in either partner, induced abortion and recurrent miscarriages. Studied paternal factors were age, tobacco use and genital tract infection. Data was computed using SPSS version 16. Significance of paternal factors was determined by Logistic Regression Analysis.

***Results*** ***:*** Total cases studied were 200, while there were 400 controls. Mean maternal age was 27.6±4.9 years in cases and 26.5±4.5 years in controls. Mean paternal age was 35.5±6.2 years in cases and 32.3±5.4 years in controls. Paternal age was >35 years in 54.5% cases and 16.8% controls. Spearman Bivariate correlation revealed paternal age > 35 years (p=0.000) and genital tract infection (p=0.043) as significant factors. Only paternal age >35 years (p=0.000) remained significant in Final Model after entering into logistic regression.

***Conclusion:*** Paternal age beyond 35 years was found to be significantly related to first trimester spontaneous miscarriages.

## INTRODUCTION

Spontaneous miscarriage is a common gynaecological condition creating an emotional crisis for the family. Around half of the spontaneous miscarriages occurring in first trimester are likely to be due to chromosomal abnormality.^1^ Chromosomal abnormality in the zygote may result from errors during gametogenesis, during fertilization or during first cellular division. Thus any such abnormality in the sperm could be a cause of spontaneous miscarriage. Advancing paternal age is being increasingly identified as one of the possible causes for such defects.^2^^-^^5^

Trends towards increasing paternal age are being observed in the UK as well as USA, due to delay in marriages for attaining better socio-economic stability.^6^ In England and Wales, mean age at childbearing rose from 26.4 years in 1974 to 29.3 years in 2002.^7^ Since 1980, birth rates in United States have increased by 40% for men aged 35 – 49 years.^8^

Advancing paternal age has been shown to result in subfertility, adverse pregnancy outcomes (miscarriage, late foetal death, preterm delivery, low birth weight), birth defects (cleft lip and palate, congenital heart defects), achondroplasia, osteogenesis imperfect , Apert’s syndrome, schizophrenia, childhood cancer (brain cancer, retinoblastoma, acute lymphoblastic leukaemia) and adult cancer (breast, prostate and nervous system).^3^ Possible mechanisms for these problems include single gene mutations, autosomal dominant diseases, structural abnormalities in sperm chromosomes (e.g., reciprocal translocations) and multiple genetic / chromosomal defects. DNA damage in sperm of men aged 36 – 57 years was found to be 3 times that of men less than 35 years.^9^

Male factors other than age e.g., occupation, environmental exposure and smoking have also been shown to influence sperm quality and therefore, affect early embryo loss.^10^ Production of reactive oxygen species e.g., by excessive stress, competitive sports, infection, alcohol, smoking, nicotine and drug abuse may result in sperm DNA fragmentation.^11^^,^^12^

The effect of female factors on adverse reproductive outcome is well known. But having said this, in majority of cases of spontaneous miscarriage the cause remains unidentified. Could paternal factors be responsible has not been studied in local population. This study, therefore, aimed to study the possible link of these factors with spontaneous miscarriage.

## METHODOLOGY

This case control study was conducted in the Department of Obstetrics and Gynaecology, Unit V / IV, Dow Medical College and Lyari General Hospital, Karachi. Duration of study was two and half years, from November, 2007 to April, 2010. Convenient sampling was used. We studied 200 cases against 400 controls. Our inclusion criteria were pregnant women between the age of 20 – 35 years, with any parity, admitted in the Department. Women with spontaneous first trimester miscarriage were taken as cases, while those admitted for delivery beyond 24 weeks of gestation were taken as controls. Gestational age was calculated from last menstrual period and confirmed by ultrasound, done from the Department of Radiology, Lyari General Hospital. Exclusion criteria were known medical illness in either partner, induced abortion and history of recurrent miscarriages.

Data were collected on pre-designed proformas by post-graduate trainees on duty. Information was obtained from the woman regarding her age, her husband’s age, parity, gestational age, ethnic group, husband’s occupation and income, tobacco / drug abuse by woman and husband and genital tract infection in husband (suggested by urethral discharge, urethral ulcer, premature ejaculation, dysuria).

All data were entered and analyzed using SPSS version 16. Means with standard deviations were calculated for numerical data, while frequencies were calculated for categorical data. Paternal factors were studied using Spearman Bivariate Correlation. Paternal age was divided into six categories i.e., 20 – 25 (reference), 26 – 30, 31 – 35, 36 – 40 and > 45 years. Maternal age, maternal tobacco / drug abuse were considered as confounders. Maternal age was divided into three categories i.e., 20 – 25 (reference), 26 – 30 and 31 – 35 years. Factors found significant were entered into logistic regression to construct the final model. Any result with p value < 0.05 was considered significant.

## RESULTS

Total Gynaecology admissions during the study period were 687. Total number of miscarriages was 363, out of which 349 (96.1%) were spontaneous miscarriages. Upon further sorting, number of first trimester spontaneous miscarriages was 240 (69%), out of which 200 cases met inclusion and exclusion criteria. They were compared with 400 controls.

Table-I shows descriptive baseline data of patients. Mean maternal as well as paternal age was higher amongst the cases as compared to controls, while parity was comparable. Mean gestational age of patients was 10.6 weeks, while controls were at term.

Paternal age was more than 35 years in 54.5% cases versus 16% controls and was more than 45 years in 1.5% and 1.2%, respectively. Majority of husbands in both groups were labourers, with income less than Rs. 5000/=. Commonest ethnic group was Balochi in more than 50%, followed by Pathans. Drug abuse history was difficult to obtain, but a substantial number of women and their husbands were using tobacco in various forms, alone or in combination (Gutka, Hukka, Pan, Beeri and Cigarette).

Table-II shows the factors studied by Spearman Bivariate Correlation. Maternal age, paternal age and genital tract infection were found to be significant risk factors. Final model (Table-III) revealed both maternal age group 31 – 35 years and paternal age > 35 years as independent risk factors.

**Table-I:** Baseline data

	*Cases*		*Controls*		*p*	*95% Confidence Interval*	
	*Mean*	*± SD*	*Mean*	*± SD*		*Lower*	*Upper*
Maternal age (years)	27.6	4.9	26.5	4.5	0.008	- 1.868	- 0.282
Parity (n)	2.5	2.1	2.7	1.9	0.176	- 0.103	+ 0.563
Paternal age (years)	35.5	6.2	32.3	5.4	0.000	- 4.131	- 2.204
Income (Pakistani Rupees)	5816.5	2685	6777.5	3030	0.000	464.3	1457
Gestational age (weeks)	10.6	1.8	37.5	1.7	0.000	26.661	27.249

**Table-II:** Factors associated with spontaneous miscarriage (Spearman Bivariate Correlation).

		*Cases*		*Controls*		*p*
		*No*	*%*	*No*	*%*	
Paternal Age (Years)	1(REF)= 20 - 25	9	4.5	46	11.5	0.000
	2= 26 - 30	47	23.5	143	35.8	
	3= 31 - 35	35	17.5	144	36	
	4= 36 - 40	88	44	48	12	
	5= 41 - 45	18	9	14	3.5	
	6= > 45	3	1.5	5	1.2	
Maternal Age (Years)	1(REF)= 20 - 25	82	41	198	49.5	0.027
	2= 26 - 30	73	36.5	136	34	
	3= 31 - 35	45	22.5	66	16.5	
Paternal tobacco / drug abuse		136	68	273	68	0.249
Maternal tobacco / drug abuse		104	52	188	47	0.951
Genital Tract Infection		13	6.5	12	3	0.043

**Table-III:** Final model (Logistic Regression).

	*p*	*OR*	*95% CI*	
			*Lower*	*Upper*
Maternal Age				
31 - 35 Years	0.003	0.360	0.185	0.702
Paternal Age				
36 – 40 Years	0.000	16.44	6.612	40.896
41 – 45 Years	0.000	13.738	4.376	43.127
> 45 Years	0.026	7.042	1.269	39.090

## DISCUSSION

The present study has demonstrated that the paternal age more than 35 years was an independent risk factor associated with spontaneous first trimester miscarriages. In order to eliminate the effect of maternal age, which is itself a known risk factor, we selected women between the age of 20 – 35 years, as this is considered to be ideal age for child bearing. Slama R, et al studied influence of paternal age on spontaneous miscarriage. They inducted women in early pregnancy and followed them to detect outcome. They found that the risk of spontaneous miscarriage with paternal age 35 or more years was 1.26 times higher (95% CI = 1.00, 1.60). The result was independent of maternal age, maternal tobacco, alcohol, caffeine and paternal tobacco use.^1^

Kleinhaus K conducted a case control study and concluded that odd ratio for spontaneous miscarriage was 1.6 (95% CI = 1.2 – 2.0, p = 0.003), for fathers aged 40 years or more. This risk was independent of maternal age and multiple other factors.^13^ Report from Reproductive Endocrinology & Infertility Committee & Collaborators states that advanced paternal age appears to be associated with increased risk of spontaneous miscarriage and some autosomal dominant conditions, autism spectrum disorder and schizophrenia. They recommend counselling of men more than 40 years of age when seeking pregnancy.^14^^,^^15^ Iwayoma and colleagues from Japan also found link between increasing paternal age and higher incidence of spontaneous miscarriage.^16^ Similar results have been reported by Veles de la Calle JF, Belloc S and Fisch H.^12^^,^^17^^,^^18^

Dane L published a systematic review on effect of paternal age on assisted reproductive outcomes.^19^ Majority of studies in this review failed to show a correlation between paternal age and spontaneous miscarriage when adjusted for maternal age, except for one study by Frattarelli et al.^20^ They concluded that overall pregnancy loss rate significantly increased with paternal age, 41.5% for > 50 years old and 24.4% for less than or equal to 50 years (p < 0.01; RR, 0.61; 95% CI, 0.45 – 0.84).

We selected the cutoff of 35 years for paternal age. Interestingly, none of the husbands in our study was less than 20 years, while more than 45 years were very few. Different authors have used different paternal ages. Kleinhaus K et al have studied various age groups and have found father’s age more than 40 years to be significantly associated with spontaneous miscarriage.^13^ Slama R has also studied age ranges and have found that risk of spontaneous miscarriage showed linear increase in the hazard of spontaneous miscarriage in male age between 20 and 45 years. They also observed that hazard ratio was highest with male age > 45 years compared with 18 – 24 years (HR = 1.87, 95% CI, 1.01 – 3.44).^1^ Others have used paternal age between 30 to more than 50 years.

Studies on paternal age and fertility suggest that male biological clock does exist. Similar to women, advancing paternal age results in negative effects on reproductive outcomes. Reduced semen volume, decline in sperm concentration, reduced sperm motility and morphology and reduced motile sperm count have been observed with increasing men’s age.^20^^-^^25^ Moreover, Luna M et al reported decline in implantation rate after assisted reproductive techniques in men > 60 years old, while Frattarelli et al and Klonoff-Cohen et al found deteriorating embryo quality.^20^^,^^23^^,^^26^ Klonoff-Cohen also found decreasing pregnancy rate with male age. Pregnancy rate was 53% for men less than or equal to 35 years, 35% for 36 – 40 years and 13% for men > 40 years.

Another observation that suggests sperm abnormalities with increasing age is the higher incidence of mutations in offsprings of aging men. Advanced paternal age is linked with poor neuro-cognitive scores, Apert’s syndrome, schizophrenia and autism.^27^^-^^30^ Ji-Yeob Choi et al in a case control study report the risk of breast cancer 1.6 fold high in offsprings of fathers aged more than 40 years than with fathers < 30 years. They suggest that the older paternal age may increase the germ cell mutation rate in the offspring.^31^

We postulate from these studies that damage to sperm accumulates over a man’s lifetime. Sperm making cells continue to divide throughout the man’s life, increasing the chances of mutations. Impaired DNA replication and repair mechanisms and increased DNA fragmentation.

DNA damage could also result from reactive oxygen species formed by alcohol, nicotine and drug abuse.^11^ We also studied these factors because of their potential to influence spontaneous miscarriage.^32^ Our study population mainly comprised of local residents from Lyari town, where nicotine use in various forms is very common, in addition to other abuse drugs, in men and women. However, only history of tobacco use could be retrieved, and none of the patients committed the use of alcohol or other abuse drugs by herself or husband. As tobacco use was almost equally common in both genders, therefore, any possible effect on spontaneous miscarriage has been nullified. Bellver J and colleagues in their study have reported that the number of cigarettes smoked by healthy men was 0 – 3 per day as compared to 0 – 20 per day in recurrent spontaneous miscarriage.^33^

Reactive oxygen species can also be formed in inflammatory processes. So we studied whether genital tract infection could also result in possible sperm damage and thereby spontaneous miscarriage. Male genital tract infection was supposed to be present on the basis of history stated by the wife and any confirmation of diagnosis could not be made. We found positive result on bivariate correlation, which did not sustain its significance on logistic regression. According to Aitken RJ’s study, male genital tract infection can result in DNA damage in male germ cells and therefore, increase the rates of miscarriage.^32^

## CONCLUSION

Paternal age more than 35 years was found to be an independent risk factor in spontaneous first trimester miscarriages.
